# Neonatal admissions and mortality: a statistical adjustment approach using birthweight-specific curves to address bias due to underreporting in 65 hospitals in four African countries

**DOI:** 10.1186/s12963-026-00496-z

**Published:** 2026-07-28

**Authors:** Lucas Malla, Siu Nam Wong, Samuel Ngwala, Evelyn Zimba, Msandeni Chiume, Norman Lufesi, Kondwani Kawaza, Honorati Masanja, Josephine Shabani, Prosper Mshana, Robert Tillya, Nahya Salim, John Wainaina, Julius Thomas, Junwei Chen, Franklin Okech, George Okello, William Macharia, Olabisi Dosunmu, Chinyere Ezeaka, Opeyemi Odedere, Morris Ogero, James H. Cross, Rebecca E. Penzias, Christine Bohne, Rebecca Richards-Kortum, Maria Oden, Eric O. Ohuma, Joy E. Lawn, with NEST360 Collaborative authorship group

**Affiliations:** 1 Department of Infectious Disease Epidemiology and International Health, London School of Hygiene & Tropical Medicine, London, UK; 2 The University of Hong Kong, Pokfulam Road & Bonham Road, Pok Fu Lam, Central and Western District, Hong Kong SAR, China; 3 Research Support Center, School of Public Health and Family Medicine, Kamuzu University of Health Sciences, Blantyre, Malawi; 4 Health Systems Impact Evaluation and Policy Department, Ifakara Health Institute, Dar Es Salaam, Tanzania; 5 Muhimbili University of Health and Allied Science (MUHAS), Dar es Salaam, United Republic of Tanzania; 6 Rice360 Institute for Global Health Technologies, Rice University, Texas, USA; 7 Kenya Medical Research Institute-Wellcome Trust, Nairobi, Kenya; 8 Aga Khan University, Nairobi, Kenya; 9 APIN Public Health Initiatives, Nairobi, Nigeria

**Keywords:** Neonatal mortality, Birthweight, Underreporting, Bayesian, Health information systems, Data quality

## Abstract

**Background:**

Underreporting of neonatal admissions and deaths – particularly among < 1000 g newborns – compromises the plausibility of in-patient neonatal mortality rate (iNMR) estimates. This limits progress tracking, since 2.3 million neonatal deaths occur annually, and 65 countries are projected to miss the Sustainable Development Goal (SDG) 3.2 target of fewer than 12 deaths per 1000 live births by 2030. Reliable iNMR estimates are essential for national and hospital-level monitoring. This study assessed birthweight-specific adjustment curves developed using simulated data, and extended the approach to routine data from 172,809 neonatal admissions across four African countries in the NEST360 network.

**Methods:**

Simulated data were generated using birthweight intervals of 500 g from 500 to 5000 g, with 1000 values per category representing 1000 hospitals. Admissions were drawn from a negative binomial distribution and mortality rates from a beta distribution based on published birthweight-specific iNMRs. Adjustment functions were developed using data from 500 hospitals without underreporting and tested on 500 with induced underreporting. This approach was then extended to real data from 65 NEST360 partnering hospitals. The adjustment functions were derived from 26 hospitals with plausible reporting (iNMR ≥ 700 per 1000 for neonates < 1000 g).

**Results:**

In simulated data, adjusted iNMRs closely matched original values, with median differences ranging from − 4 to 9 deaths per 1000 across birthweight categories. In NEST360 dataset, unadjusted iNMRs were 121 (range 13–272) in Nigeria, 128 (60–214) in Tanzania, 132 (42–234) in Malawi, and 135 (60–250) in Kenya per 1000 admissions. After adjustment, estimates increased to 162 (127–333), 183 (128–233), 174 (120–268), and 172 (123–261), respectively. Variability across hospitals decreased, with standard deviation reducing from 13 to 5 deaths per 1000.

**Conclusions:**

Birthweight specific adjustment functions provide a practical and scalable approach to improving the plausibility of iNMR estimates where underreporting is common. These methods can be integrated into routine systems (such as Electronic Medical Record Systems and DHIS2) where key data are available, with modest computational demands. Their use can strengthen mortality surveillance and support more reliable policy and planning, while contributing to progress toward SDG 3.2, contingent on continued improvements in data quality and system capacity.

**Supplementary Information:**

The online version contains supplementary material available at https://doi.org/10.1186/s12963-026-00496-z.

## Key Findings

WHAT WAS KNOWN?

Underreporting of neonatal admissions and mortality is common in many countries, particularly for neonates <1000g, leading to underestimation of in-patient neonatal mortality rates (iNMRs). This has important implications for comparing between hospitals or over time, evaluation of interventions, as well as planning resource allocation and tracking progress towards SDG 3.2 for newborn survival.

WHAT WAS DONE THAT IS NEW?

Based on context-specific data, a simulation-based approach was used to develop birthweight-specific adjustment functions to account for underreporting of admissions and in-patient neonatal deaths. Various levels of underreporting were then introduced, and adjustment models were re-fitted to estimate the original simulated values.

The approach was extended to adjust for underreporting of admissions and deaths in 65 hospitals partnering with NEST360 in four countries (Kenya, Malawi, Nigeria, and Tanzania). Adjustment curves were developed using data from 26 hospitals with plausible mortality rates (≥700 deaths per 1000 live births for neonates <1000g) to create robust birthweight-specific adjustment functions. These functions were then applied across data from all 65 hospitals to adjust for underreporting in a dataset of 172,809 baseline in-patient neonatal mortality admissions.

WHAT WAS FOUND?

Applying birthweight-specific adjustment functions to simulated data demonstrated the statistical approach to be robust in adjusting for underreporting, since adjusted neonatal iNMRs closely matched the original simulated values ("truth") even at varying levels of underreporting.

Using a dataset of 172,809 admissions from 65 hospitals partnering with NEST360, unadjusted iNMRs were 121 deaths per 1000 admissions in Nigeria, 128 per 1000 in Tanzania, 132 per 1000 in Malawi, and 135 per 1000 in Kenya. Birthweight-specific adjustment functions were developed based on 26 hospitals with plausible mortality rates (≥700 deaths per 1000 live births for neonates <1000g). After applying adjustment curves, the iNMRs increased to 162 deaths per 1000 admissions in Nigeria, 183 per 1000 in Tanzania, 174 per 1000 in Malawi, and 172 per 1000 in Kenya. As expected, adjusted iNMRs were higher and reduced inter-hospital variability.

WHAT NEXT?

This adjustment method may be useful more widely, including in LMIC national routine health data, which is now the main source of neonatal data, given recent defunding of Demographic Health Surveys.

Share learning to improve capture of all admissions and deaths in routine data systems, such as increased communication that all live-born babies are to be registered, irrespective of gestational age or weight, regular data audits, and educating/mentoring to promote accurate and complete reporting.

**Supplementary Information:**

The online version contains supplementary material available at https://doi.org/10.1186/s12963-026-00496-z.

## Background

Countries in all regions have made substantial progress in reducing under-five mortality since 1990, largely due to targeted public health interventions, and global initiatives aimed at child survival [1]. However, the reduction in neonatal mortality – deaths occurring within the first 28 days of life – has been much slower, particularly in sub-Saharan Africa. Neonatal deaths are 2.3 million per year worldwide now account for almost half of under-five mortality, with 65 countries off track for the 2030 Sustainable Development Goal of fewer than 12 deaths per 1000 live births by 2030 [2]. A critical challenge in tackling neonatal mortality is the gap in the availability and quality of specialised care for small and sick newborns. While efforts have been made to improve basic newborn care, such as neonatal resuscitation and Kangaroo Mother Care (KMC), hospitals in Africa often have limited infrastructure and capacity to provide neonatal care, particularly for preterm babies who require respiratory support [3]. As a result, over one million newborns die needlessly each year in Africa, with the majority of these deaths caused by preventable conditions such as infections, birth complications, and prematurity [4–7].

Addressing this gap in the quality of care for small and sick newborns requires a concerted effort to scale up interventions within hospitals, focusing on improving both availability and quality. This is the impetus behind the Newborn Essential Solutions and Technologies (NEST360) alliance, which was launched in Malawi, Tanzania, Kenya, and Nigeria during phase 1 (2019–2023), and Ethiopia during phase 2 (2024–2028). NEST360 builds on key lessons from Malawi’s national scale-up of bubble continuous positive airway pressure (CPAP), which demonstrated that effective neonatal respiratory support can be delivered at district hospital level when appropriately systematised. The Malawi experience showed that improved survival among very low birth weight babies, particularly those weighing 1.0–1.3 kg, was achievable through early initiation of CPAP outside tertiary centres, supported by structured training of non-specialist providers, reliable oxygen delivery systems, and standardised clinical protocols. It also highlighted the importance of decentralising care to reduce delays in treatment initiation and the need for strong implementation support to ensure consistent use and maintenance of devices [8]. These lessons informed NEST360’s approach to device adaptation for low-resource settings and its emphasis on scalable training, mentorship, and integrated systems strengthening across district hospitals.

NEST360 is a collaboration of 23 partner organizations, 18 of which are based in Africa, working alongside governments to implement the initiative’s package of interventions across more than 100 hospitals. The NEST360 approach includes innovative medical devices, healthcare worker training, mentorship, quality improvement initiatives, and improved health information and data systems to support better newborn care. By supporting African governments in scaling up these interventions, NEST360 aims to significantly reduce in-patient neonatal deaths across the five participating countries.

The impact of NEST360 implementation will be evaluated by examining changes in in-patient neonatal mortality rates (iNMRs) over time. A critical challenge in accurately measuring the impact of the initiative is underreporting of neonatal admissions and deaths [9]. Underreporting is a widespread problem in both high- and low-income countries, and it can significantly distort mortality estimates. For example, classic studies have documented underreporting in vital registration systems in the United States [10], and recent reports highlight similar issues in countries like Denmark, despite their robust health data system [11]. In lower-income settings, including many countries in Africa and Asia, underreporting is even more prevalent due to weaker health information systems and inconsistencies in data collection and reporting practices [12–14].

Several factors contribute to the underreporting of neonatal admissions and deaths, and these must be addressed to ensure plausible estimates of iNMRs and to properly assess NEST360’s impact. Some of these factors include:

- **Lack of clarity over registration guidelines for live births**: One of the key reasons for underreporting stems from a lack of clear guidelines on registration criteria. For example, health professionals may misinterpret or misapply guidelines for distinguishing stillbirths from neonatal deaths [15]. Such misclassification reduces the accuracy of reported iNMRs and results in the underreporting of deaths of the most vulnerable neonates.
- **Perceptions of viability**: Healthcare workers’ perceptions of viability at the limits of prematurity vary across settings and are often shaped by local survival rates and resource constraints. Evidence from low-resource neonatal units indicates that clinicians may perceive extremely preterm babies as having very low chances of survival, which influences decisions regarding the initiation and intensity of care [16, 17]. In settings where documentation systems are weak and clinical workloads are high, this may also translate into lower prioritisation of recording admissions and outcomes for these babies, contributing to incomplete data capture and potential underreporting of neonatal deaths.
- **Reporting incentives and disincentives**: Reporting practices can also be influenced by hospital-level incentives and disincentives. Hospitals may face pressure to report lower mortality rates to avoid negative scrutiny or penalties. For example, hospitals with high mortality rates may be perceived as underperforming, which could result in reduced funding or other punitive measures. In response, hospital staff may underreport neonatal deaths to present a more favourable picture of their performance. Conversely, in settings where there is less emphasis on or fewer resources for data collection, hospitals may report fewer neonatal admissions and deaths simply because of limited capacity to track and document all cases [18, 19]. Both types of incentives – whether aimed at avoiding penalties or reflecting actual limitations – contribute to the underreporting problem and affect the quality of neonatal mortality data.
- **Lack of accountability**: Weak oversight and inadequate accountability mechanisms also contribute to incomplete reporting. In some hospitals, limited supervision, a lack of routine data audits, and minimal consequences for inaccurate or missing documentation mean that neonatal admissions and deaths may not be systematically recorded. Without clear accountability structures, healthcare workers may not prioritise comprehensive data entry, leading to gaps in reporting that undermine accurate mortality assessments [20].

Without addressing these issues of underreporting, it is impossible to obtain or estimate the true burden of neonatal mortality. Accurate data on neonatal admissions and deaths are critical not only for evaluating the success of NEST360 but also for understanding broader trends in neonatal health across LMICs.

**Aim and Objectives**.

The primary aim of this paper was to develop and apply adjustment functions based on birthweight-specific distributions to account for the underreporting of neonatal admissions and deaths, thereby generating more plausible estimates of in-patient neonatal mortality rates (iNMRs). This goal was addressed through two specific objectives:

1. **Feasibility of adjusting for underreporting using simulated birthweight-specific curves**: This objective involved developing and applying birthweight-specific admissions and mortality curves to simulated data. The goal was to evaluate whether these curves effectively adjusted for (different levels) of underreporting. This step served as a proof of concept, demonstrating how these adjustments led to more plausible iNMR estimates.
2. **Application of the validated approach to NEST360 partnering hospital data**: Building on the insights gained from the simulation, birthweight-specific adjustment functions were developed and applied to empirical data from phase one NEST360 partnering hospitals. These functions aimed to adjust for underreporting in baseline iNMRs, ensuring that the adjusted estimates more plausibly reflected the actual burden of neonatal mortality within these hospitals.

## Methods

### Overview

To address the first objective, we simulated birthweight-specific admissions and in-patient neonatal mortality data across nine predefined birthweight categories for 1000 hypothetical hospitals. Bayesian regression models were developed using data from the first 500 hospitals to generate adjustment functions for admissions and mortality. These functions were then applied to the remaining 500 hospitals, in which underreporting had been introduced randomly. For the second objective, we applied this adjustment approach to data from 65 hospitals implementing with NEST360. After identifying 26 hospitals with more plausible mortality data, we developed birthweight-specific adjustment functions. These were thereafter applied across all hospitals to adjust for underreporting and estimate plausible in-patient neonatal mortality rates at both hospital and country levels.

**Objective 1: Feasibility of adjusting for underreporting using simulated birthweight-specific curves**.

In this objective, admissions and mortality data were simulated by randomly sampling from predefined distributions of birthweight in 500 g intervals between 500 and 5000 g. These categories were: <1000 g, 1001–1500 g, 1501–2000 g, 2001–2500 g, 2501–3000 g, 3001–3500 g, 3501–4000 g, 4001–4500 g, and 4501–5000 g. For every birthweight category, we generated 1000 random values to represent data for 1000 hospitals.

### Simulating mortality and admissions data

We simulated admission counts using a negative binomial distribution to allow for varying levels of dispersion, parameterised such that the variance is µ + µ²/θ. Mean admission numbers (µ) for simulations were chosen arbitrarily and randomly within the limits that defined a bell-shaped distribution across the birthweight categories. A baseline dispersion parameter (θ = 100) was used to approximate near-Poisson variability for groups with relatively stable admission volumes. To introduce heterogeneity across birthweight groups, we applied multiplicative scaling factors to θ (ranging from 4 to 16), generating a structured range of dispersion scenarios. These values were randomly selected to produce plausible variability relative to the mean and to assess the robustness of findings under differing dispersion conditions.

For mortality, we aimed to generate mortality rates expressed as the number of neonatal deaths per 1000 admissions across different birthweight categories. As mortality rates range between 0 and 1000, we assumed a beta distribution (bounded on [0,1]) as a plausible data-generating process for simulation [21]. We derived input parameters from published birthweight-specific iNMRs, synthesised from two systematic reviews (Blencowe et al., 2013; and the 2020 systematic review of facility-based neonatal outcomes) and selected large (multi) country cohort studies from LMICs and comparable high-income neonatal care settings [11, 22–24] (detailed in eTable 1 – all eTables and eFigures are presented in the supplementary material). For synthesis, we extracted reported birthweight-specific iNMRs from each study and harmonised them into standard birthweight categories. We then summarised values within each category using median mortality rates and ranges across studies, without applying statistical weighting due to heterogeneity in study design, setting, and level of neonatal care. Median iNMRs declined with increasing birthweight, from 827 per 1000 admissions among neonates weighing 500–999 g to 90 per 1000 in the 2500–3999 g group, with a slight increase among those > 4000 g (eTable 2). These category-specific medians were used to parameterise the beta distributions in the simulation model.

### Constructing admissions and mortality birthweight-specific adjustment curves using simulated data for the first 500 hospitals

For birthweight (BWT) category * b*, admission models were expressed as:

$$\begin{gathered}\:{Admission}_{BWT=b}\\=\:{\beta\:}_{0}+\:\sum_{j=1}^{8}{\beta\:}_{j}*{Admission}_{BWT\ne\:b}+\:\epsilon\end{gathered}$$

where $\:{\beta\:}_{0}$ is the intercept with $\:{\beta\:}_{j}$ (j = 1…8) representing parameters associated with admissions data in the remaining eight birthweight categories and *ε* the error term. In each of these models, admission numbers were modelled while assuming a negative binomial distribution. The modelled numbers were used to estimate the proportion of admissions across the various birthweight categories, serving as the basis for adjustments.

In developing adjustment curves for mortality data, beta regression models were specified separately for babies weighing < 2500 g and ≥ 2500 g using simulated data for the first 500 hospitals. We applied this stratification based on the established low birthweight threshold (< 2500 g), which is widely used to distinguish neonatal risk profiles [25]. The iNMR models were therefore of the form:

$$\begin{gathered}\:{iNMR}_{BWT=b}\\=\:{\beta\:}_{0}+\:\sum_{j=1}^{n}{\beta\:}_{j}*{iNMR}_{BWT\ne\:b}+\:\epsilon\end{gathered}$$

where n is the number of birthweight categories; *n* = 4 and 5 for birthweight < 2500 g and ≥ 2500 g respectively. Beta distribution with a logit link function was used in modelling iNMR for each birthweight category [21].

All admissions and mortality models were fitted within a Bayesian framework. This approach is advisable because future applications would potentially involve data with small sample sizes or challenges related to data sparsity [26, 27]. For all parameters, weakly informative uniform priors were specified, with models fitted using three chains and 30,000 iterations, half of which were used as burn-in for each model. Trace plots were used to assess how well the posterior distributions were explored [28]. Convergence was evaluated using the potential scale reduction factor (PSRF), which measures both between-chain and within-chain variability in the sampled parameter values. A PSRF value of 1 indicates adequate convergence [29]. In addition, the effective sample sizes (ESS) were also assessed.

Model fit was assessed using posterior predictive checks by comparing observed hospital-level admissions and iNMR with posterior predictive summaries based on 1000 simulated draws from the posterior predictive distribution. For each hospital, the posterior predictive mean was computed by averaging across all draws. These summaries were compared with observed hospital-level values to assess how well the model reproduced patterns across hospitals. The evaluation focused on examining the distribution of admissions and iNMR within birthweight categories, comparing observed category-specific hospital-level means with corresponding posterior predictive mean estimates, and assessing whether the model reproduced overall distributional patterns and variability across hospitals and say birthweight categories.

### Introducing underreporting to second half of the simulated data

For the second set of 500 hospitals, we randomly introduced underreporting across all birthweight categories. The underreporting factors were drawn from a uniform distribution, with values ranging between 7% and 55%, based on previous literature on neonatal death underreporting. This range reflected data from both developed and developing countries, including Pakistan, Brazil, and several Eastern European nations [30–32]. The uniform specification was used to represent uncertainty regarding the exact form of the distribution while ensuring that simulated values remained consistent with empirically observed bounds.

### Application of developed curves to the derived data with underreporting from step 3

The estimated adjustment functions were applied to adjust for underreporting and data gaps in the birthweight-specific admissions and mortality rates for the remaining 500 hospitals.

### Assessing the performance of the adjustment functions

For each of the nine birthweight categories, we compared the number of admissions and neonatal mortality rates between simulated values (‘the truth’) and adjusted values, accounting for underreporting. These differences were visualised using boxplots. We then assessed the similarity between hospital-level in-patient neonatal mortality rates before and after adjusting for underreporting. To quantify this, we calculated the absolute standardised mean difference (SMD) between the two sets of hospital-level iNMRs, considering an SMD below 10% as satisfactory [33]. Finally, we used Welch’s t-test to determine the statistical (non) significance of differences in these hospital-level iNMRs.

**Objective 2: Application of the approach of using birthweight-specific distributions for adjustments to hospital data with NEST360**:

### NEST360 neonatal in-patient database and data

Together with partners, the NEST360 team developed a neonatal in-patient database in REDCap [34]. The database hosts a core dataset and consolidates data from at least 100 hospitals across the implementing countries. Data capture forms in REDCap were structured to align with the following six broad categories: Birth details and maternity history, admission details, complications and observations, interventions, discharge outcomes and diagnosis or cause of death. Data capture, collation, transfer, and quality assessment are detailed in Cross et al. (2022) [35] and briefly highlighted as follows:

- **Data capture**: Data are abstracted retrospectively from patient files by trained data clerks based in the hospitals. Data abstraction and entry into REDCap electronic forms are carefully guided by well-defined standard operating procedures (SOPs), which are used in all the hospitals to ensure consistency and accuracy. In Kenya, the Clinical Infection Network (CIN) already collects neonatal in-patient data, and in Tanzania, one hospital captures neonatal in-patient data through Electronic Medical Record system. The NEST360 team leveraged on these existing systems to limit duplication of efforts.
- **Data collation and transfer**: Data are first collated in each country’s server, then deidentified and digitally transferred to a central server at LSHTM using secure Application Programming Interfaces (APIs) [36]. The transfer is in accordance with a data sharing agreement for NEST360 participating countries.
- **Data quality assessment (DQA)**: Within the central server, variables are further processed, and data quality are assessed. Processing of variables, for example, involves recoding and mapping of Kenya’s CIN variables to match those in NEST360’s data dictionary. While assessment of data quality involves checking of completeness of key variables, adherence to skip logics, and checking of out-of-range values. The algorithms for examining these three data quality elements are directly derived from the data dictionary through metaprogramming in R statistical software [37]. The outputs of this process are presented as reports per hospital summarising the extent of deviation from the rules defined in the data dictionary. These reports are generated in real-time and are accessible to the country teams for timely correction of errors and reconciliation of any inconsistencies.

This analysis uses phase 1 data from 65 hospitals implementing with NEST360 in Kenya, Malawi, Tanzania, and Nigeria.

### Conducting within data adjustment using birthweight specific admissions and iNMR distributions for NEST360 partnering hospitals

#### Examining evidence of underreporting in hospitals partnering with NEST360

We first analysed the NEST360 baseline mortality dataset to examine evidence for underreporting in the lowest birthweight categories. This was done using the distribution of admissions and mortality across birthweight categories. Additionally, we assessed unadjusted in-patient neonatal mortality rates (iNMR) at the hospital and country levels for further evidence of underreporting.

#### Developing birthweight specific admission and iNMR adjustment functions using NEST360 partnering hospitals with more plausible data

Based on exploratory analysis in step 1, hospitals with more plausible data were selected for the development of admission and iNMR adjustment functions. A conservative selection criterion of iNMR ≥ 700 deaths in every 1000 admissions for babies weighing < 1000 g was used. This cut-off was considered conservative as mortality in this group without full intensive care is known to be at least 800 per 1000 [11, 38]. Further, studies have indicated that iNMRs ≥ 750/1000 in the < 1000 g birthweight category potentially suggest appropriate reporting of neonatal deaths (for example, see [10]). The ≥ 700/1000 threshold was therefore used as a pragmatic criterion to balance data plausibility with sufficient sample size for model estimation, while remaining consistent with established evidence on expected inpatient mortality levels. Development of adjustment functions for admissions and mortality data used similar negative binomial and beta regression models and approach explained in step 2 of objective 1.

#### Applying the estimated adjustment functions to all NEST360 partnering hospitals to estimate baseline iNMRs

The estimated adjustment functions were applied to adjust for underreporting and data gaps in the remaining hospitals’ birthweight-specific admissions and mortality rates. Following this, we estimated hospital-level, country-level, and overall in-patient neonatal mortality rates (iNMRs) using the adjusted birthweight-specific admissions and iNMR data from each hospital.

#### Sensitivity analysis

We also considered a higher threshold of iNMR ≥ 750/1000 for babies < 1000 g, using data from hospitals that meet this threshold to develop adjustment functions. These functions were then applied to estimate the adjusted iNMRs at both the hospital and country levels.

## Results

**Objective 1: Adjusting for underreporting using simulated birthweight-specific curves**

### Summary of simulated data for 1000 hospitals

**Fig. 1:**
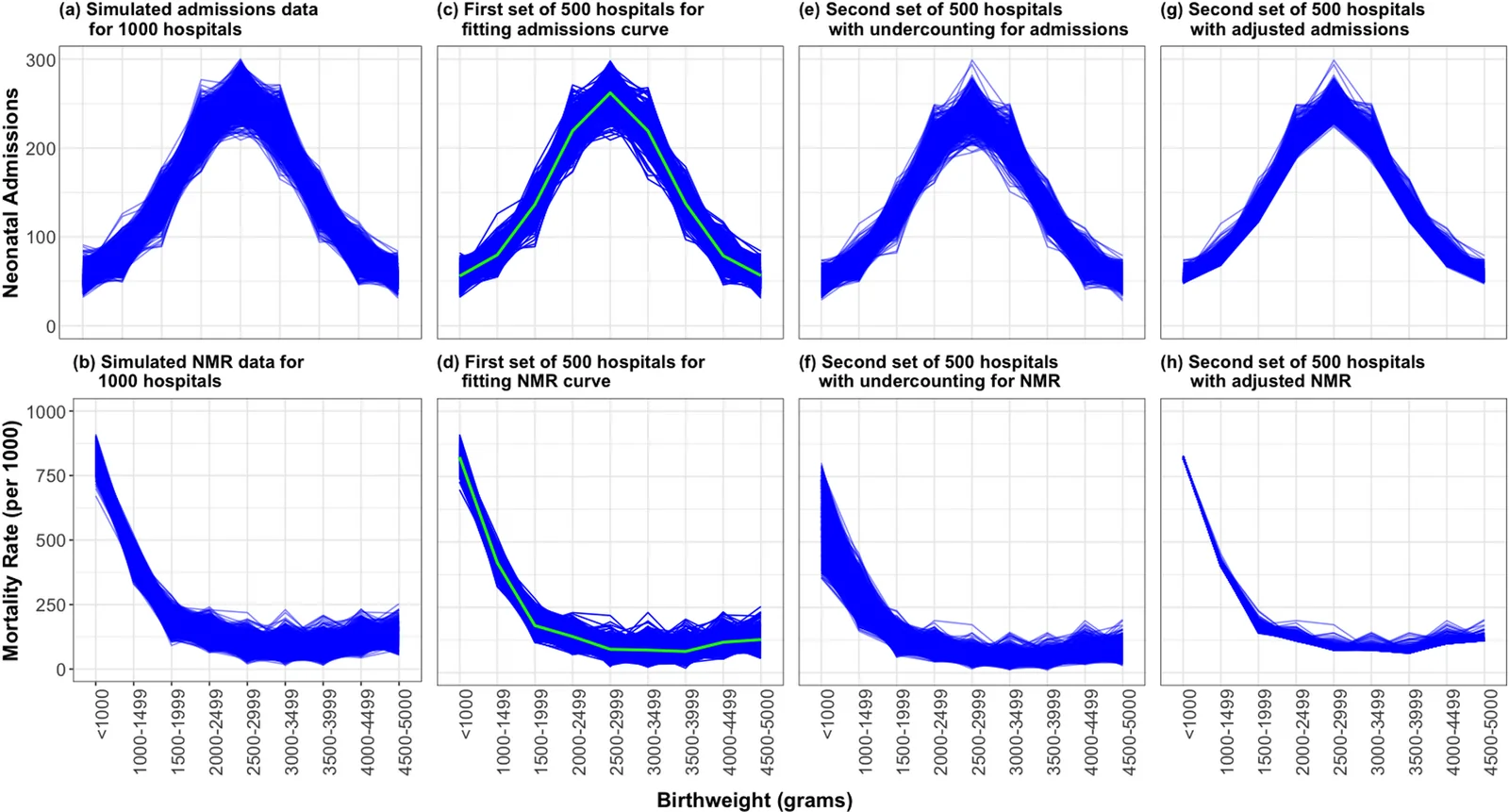
Simulated admissions and mortality data (before and after adjustment)

Simulated data representing admissions and iNMRs across 1000 hospitals are shown in Fig. 1a and b and summarised in supplementary eTable 3. The < 1000 g birthweight category had the lowest median admissions at 57, with a range from 28 to 84, indicating considerable variability in admissions across hospitals. In contrast, the 2501–3000 g category had the highest median admissions at 263, with a range from 214 to 323, reflecting more consistent admissions across hospitals. The 1501–2000 g category had a median of 136, with a range from 96 to 190, while the 4501–5000 g category showed the smallest spread in admissions, with a median of 55 and a range from 29 to 92.

Regarding mortality rates per 1000 admissions, the < 1000 g category showed the highest median at 827, with a range from 671 to 911, reflecting substantial variation in in-patient mortality between hospitals. The 1001–1500 g category showed a median in-patient mortality rate of 420, with a range from 329 to 520. As birthweight increased, in-patient mortality rates decreased: the 1501–2000 g category had a median of 178, with a range from 91 to 286, while the 2001–2500 g category had a median of 137, with a wide range from 63 to 241. In the higher birthweight categories, in-patient mortality rates were relatively lower, with the 2501–3000 g category showing a median of 85 (range 20–219), and the 3001–3500 g ,3501–4000 g, 4001–4500 g, and 4501–5000 g categories all had medians between 83 and 126.

### Fitted adjustment curves using simulated data for the first 500 hospitals

The posterior predictive mean estimates for admissions and iNMR for birthweight categories are shown in green in Fig. 1c and d. For a subset of 30 hospitals (see eFigs. 1 and 2), observed hospital-level data were compared with posterior predictive mean estimates based on 1000 posterior draws to assess model performance. The posterior predictive mean estimates broadly reproduced the observed patterns for both admissions and iNMR across birthweight categories. Overall, the models captured the general pattern and level of the observed data across hospitals and birthweight categories, indicating reasonable agreement between observed and model-generated summaries.

### Adjustment of underreporting in the second set of 500 hospitals

The data demonstrated that the adjustment process significantly improved the plausibility of iNMRs across all birthweight categories (Table 1; Figs. 1e – h). For instance, in the < 1000 g category, the median iNMR in the simulated data was 827 per 1000 admissions, which was substantially reduced to 573 per 1000 in the underreported data. After adjustment, the median iNMR increased to 825 per 1000, bringing it much closer to the simulated value. Similarly, in the 1001–1500 g category, the median iNMR in the simulated data was 420 per 1000, while it dropped to 286 per 1000 in the underreported data and then adjusted to 415 per 1000, closely mirroring the simulated data. This trend of the adjusted data aligning closely with the simulated values was seen throughout the birthweight categories, suggesting that the adjustment process effectively addressed underreporting and provided a more accurate reflection of neonatal mortality rates.

**Table 1:** Summary of simulated data for the second set of 500 hospitals, with underreporting and after adjustment (median [hospital minimum - maximum])

Birthweight	Admissions			iNMR per 1000 admissions		
	Simulated data	Data with undercounting	Data after adjustment	Simulated data	Data with undercounting	Data after adjustment
0–1000 g	57 [28–84]	52 [26–78]	54 [47–78]	827 [671–911]	573 [362–803]	825 [820–829]
1001–1500 g	78 [48–117]	72 [45–107]	75 [66–107]	420 [329–520]	286 [159–456]	415 [406–456]
1501–2000 g	135 [96–181]	126 [90–167]	130 [117–167]	178 [91–286]	121 [59–237]	170 [150.8–237.3]
2001–2500 g	220 [176–264]	205 [162–249]	207 [187–249]	137 [64–241]	95 [42–199]	130 [121–199]
2501–3000 g	263 [214–314]	243 [198–288]	248 [219–288]	85 [20–219]	59 [14–183]	90 [85–183]
3001–3500 g	221 [179–268]	204 [166–252]	208 [183–252]	83 [14–230]	56 [11–154]	89 [85–154]
3501–4000 g	137 [103–185]	127 [93–173]	130 [114–173]	78 [16–209]	53 [10–153]	80 [74–153]
4001–4500 g	78 [48–115]	72 [44–106]	75 [66–106]	116 [44–232]	82 [31–199]	115 [111–199]
4501–5000 g	55 [29–82]	51 [28–76]	53 [46–76]	127 [55–253]	84 [27–203]	126 [123–203]

### Overall performance of adjustment functions on the simulated data

**Fig. 2:**
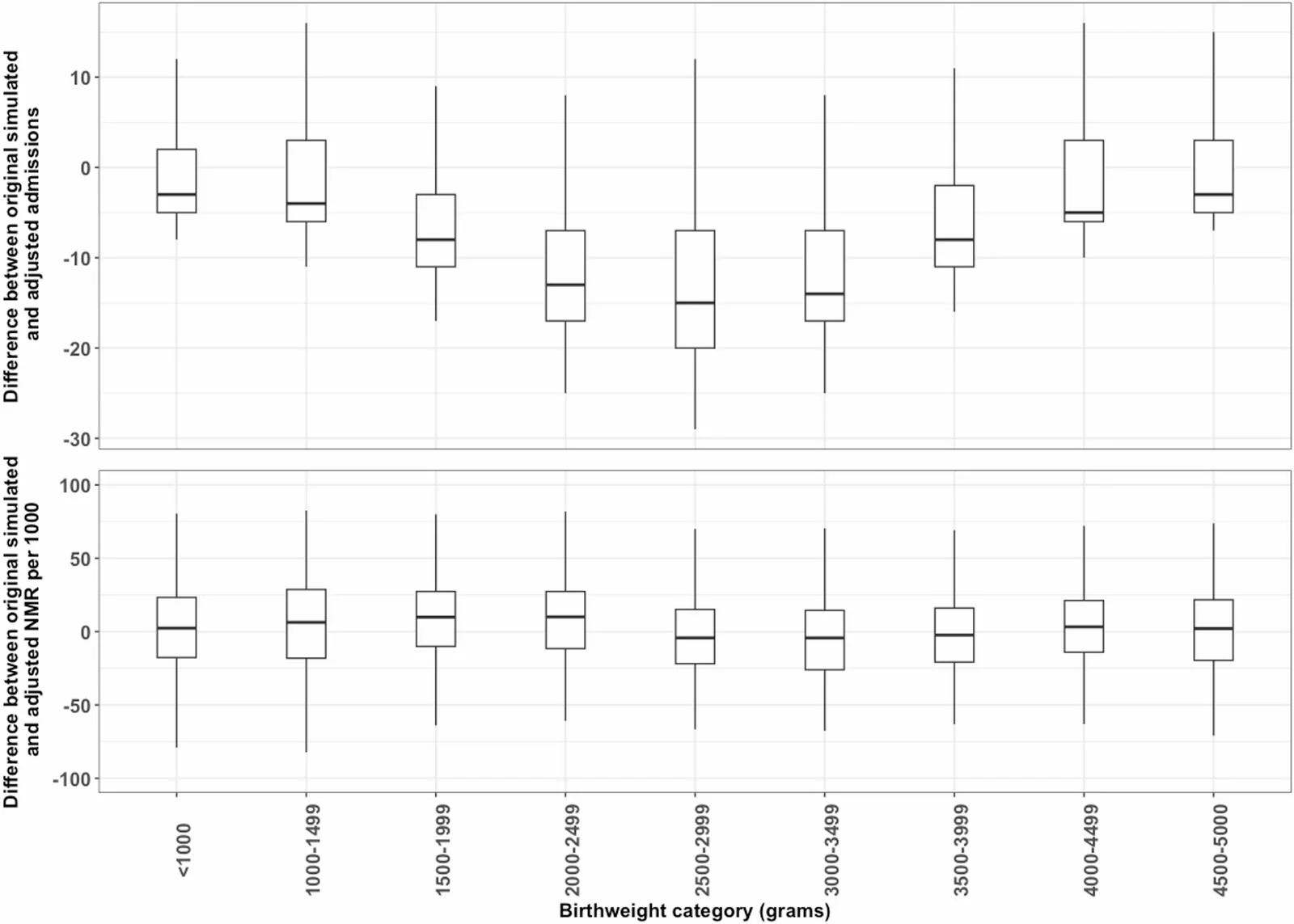
Differences in admissions and iNMR between the originally simulated and adjusted iNMR for the second set of 500 hospitals

Figure 2 shows the distribution of hospital-level differences in admissions and iNMRs between the original simulated dataset and the dataset after applying the adjustment functions for birthweight categories. The median differences are close to zero, indicating strong agreement between the two datasets. Median differences in admissions ranged from 5 to 15, while median differences in iNMRs ranged from − 4 to 9 deaths per 1000. For both admissions and iNMRs, the distribution of differences spans zero, indicating less meaningful deviation between the simulated and adjusted datasets. In addition, the standardised mean difference in hospital-level iNMR (computed from pooled data across all birthweight categories) before and after adjustment was 2.5%, which is well within the acceptable range of ± 10% of standardised mean difference. A t-test further confirmed the absence of a statistically significant difference between the two sets of hospital-level iNMRs (mean difference = 0.31 per 1,000; 95% CI: −0.92 to 1.57; p-value = 0.6036).

**Objective 2: Application of birthweight-specific adjustment functions to NEST360 hospital-level data**.

### Baseline data overview

Baseline data were obtained for 65 hospitals with a total of 172,809 admission records, which were retrospectively abstracted from January 2019 for these hospitals. Table 2 summarises per country the number of baseline records that were used for this analysis. The duration of baseline data varied for each hospital and depended on when NEST360 started implementation. Eleven hospitals had baseline data for 7–21 months, thirty-one had data for > 21–28 months, and twenty-three had data for > 28 months. About three-quarters of the admitted neonates were born within the units.

**Table 2:** NEST360 baseline neonatal in-patient data by country

Country	Number of hospitals	Baseline admission records
Malawi	36	69,938
Kenya	13	53,831
Tanzania	7	39,048
Nigeria	9	9,992
**Total**	**65**	**172**,**809**

All analyses have been undertaken with the hospital as the unit of analysis

The primary causes of mortality were prematurity, intrapartum-related conditions, and infection. Together, these three conditions account for 43%, 24%, and 12% of total deaths, respectively, underscoring their dominant contribution to the overall mortality burden and highlighting their central importance for targeted interventions within this context.

### Conducting within data adjustment using birthweight specific admissions and iNMR distributions for NEST360 partnering hospitals

#### Evidence of underreporting in NEST360 partnering hospitals

**Fig. 3:**
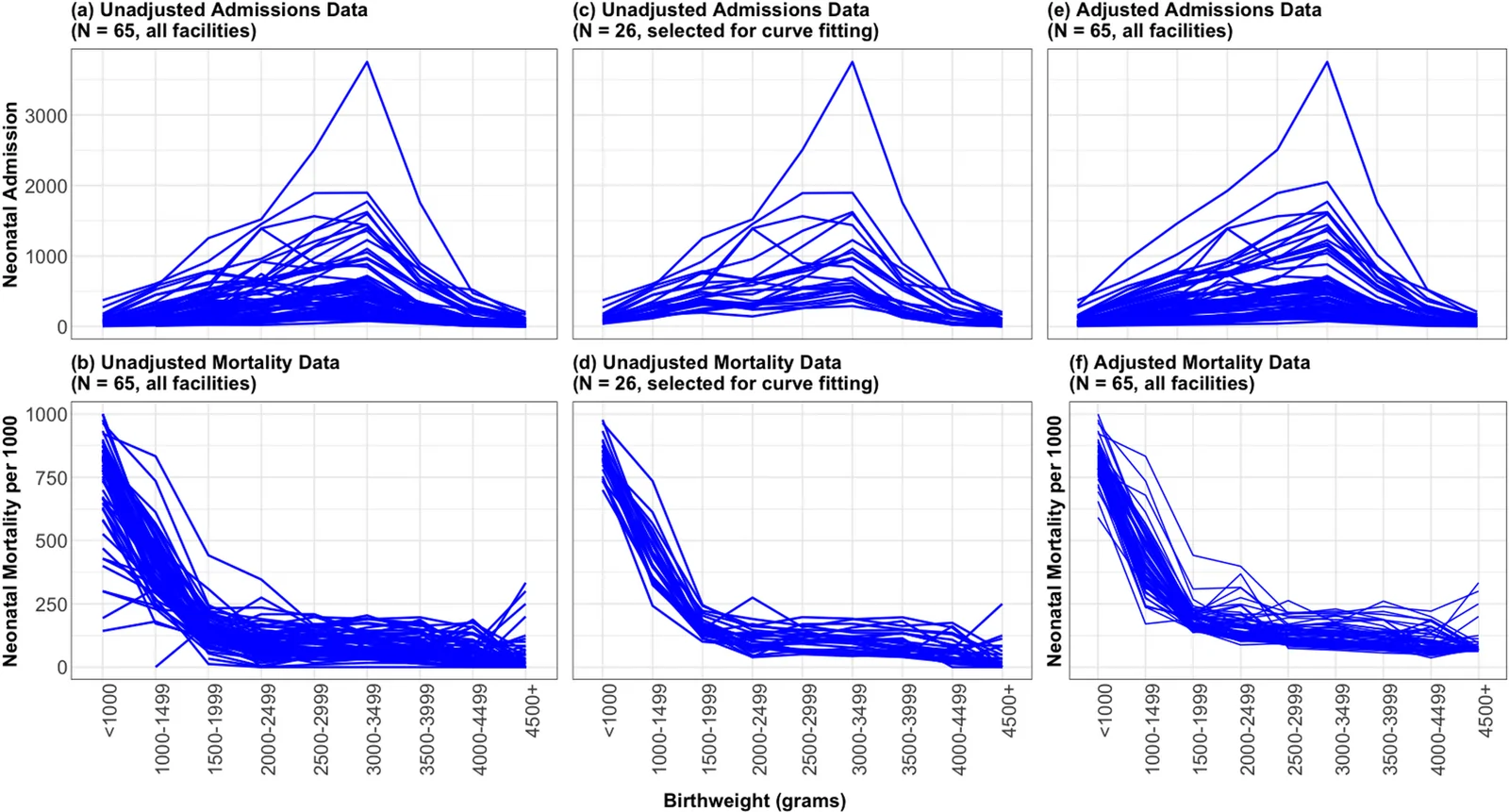
hospitals

Figure 3a and b show the distributions of unadjusted baseline birthweight-specific admission and iNMR curves for 65 hospitals. Notably, one hospital reported zero deaths per 1,000 admissions among neonates weighing < 1000 g, despite mortality in this group being known to be high, highlighting clear undercounting in this high-risk category. This pattern was not limited to the smallest babies; underestimation of in-patient neonatal mortality rates was also observed across other birthweight categories.

**Fig. 4:**
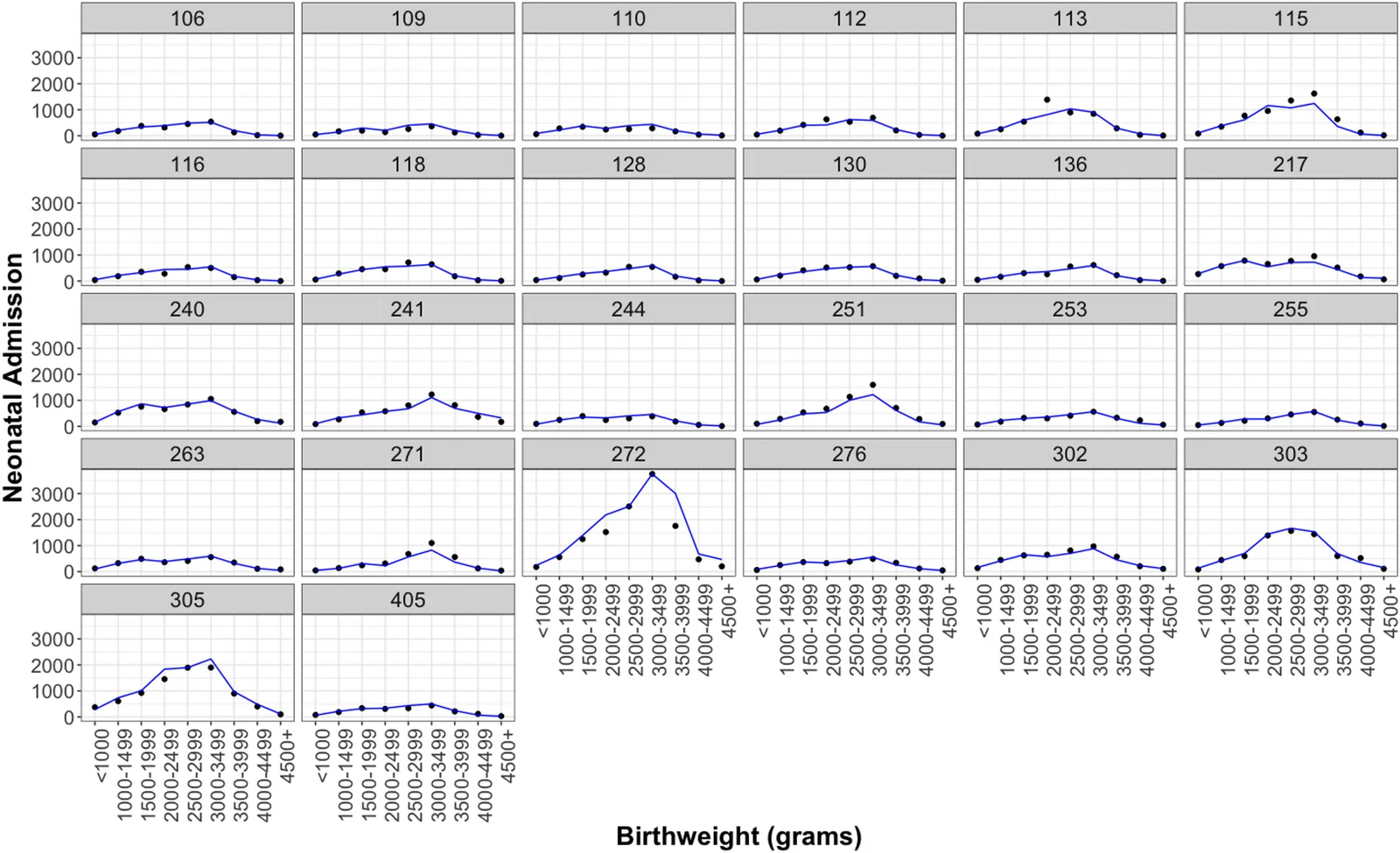
Distribution of admissions and BWSM rate for NEST360 partnering hospitals with plausible BWSM rate of ≥ 700/1000 in neonates with birthweight < 1000 g across the four countries (*N* = 26 hospitals)

At the hospital level, several unadjusted birthweight-specific admission and iNMR estimates appeared substantially underestimated. Most hospitals reported unexpectedly low baseline iNMRs given the level of care available in these settings; specifically, 21 of 65 hospitals reported unadjusted in-patient NMRs below 100 deaths per 1,000 admissions. There was also considerable heterogeneity, with hospital-level estimates ranging from 13 to 272 deaths per 1,000 admissions (Table 3).

**Table 3:** Unadjusted and adjusted (based on iNMR ≥ 700/1000 threshold) baseline iNMR estimates for 65 NEST360 partnering hospitals by country

Country	iNMR/1000 admissions (hospital range)		Number of hospitals with iNMR (per 1000)							
	Unadjusted	Adjusted	> 200		> 150–200		> 100–150		<=100	
			Unadj	Adj	Unadj	Adj	Unadj	Adj	Unadj	Adj
Kenya	135 [60–250]	172 [123–261]	3	4	2	5	4	4	4	0
Malawi	132 [42– 234]	174 [120–268]	3	8	11	20	12	8	10	0
Nigeria	121 [13–272]	162 [127–333]	1	1	0	5	5	3	3	0
Tanzana	128 [60–214]	183 [128–233]	2	3	0	3	1	1	4	0
**Total**	**131** **[13–272]**	**175** **[120–333]**	**9**	**16**	**13**	**33**	**22**	**16**	**21**	**0**

At the country level, the unadjusted average iNMRs were similar: 121 per 1,000 in Nigeria, 128 per 1,000 in Tanzania, 132 per 1,000 in Malawi, and 135 per 1,000 in Kenya.

#### Birthweight specific admission and iNMR adjustment functions fitted using NEST360 partnering hospitals with more plausible data

**Fig. 5:**
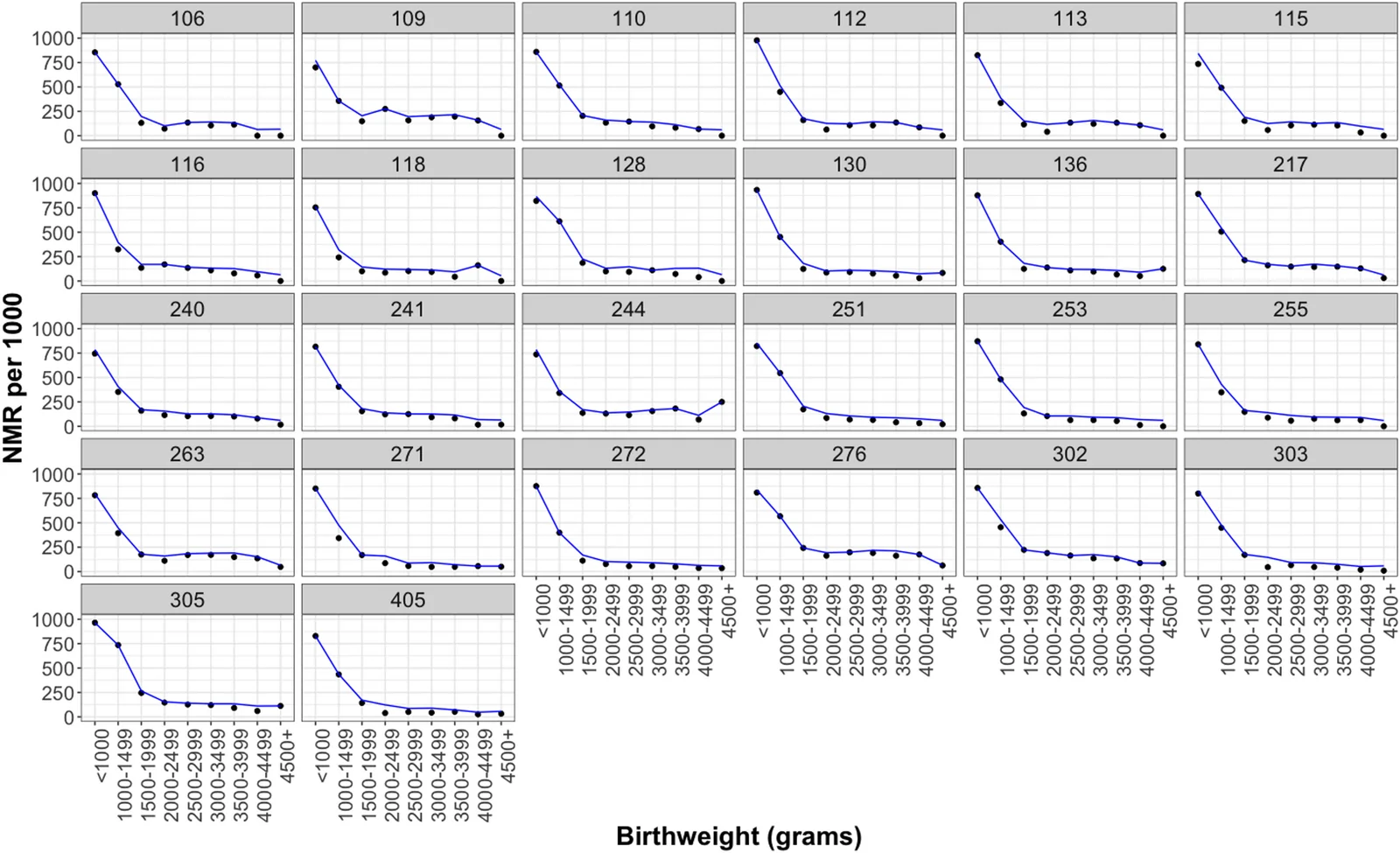
Distribution of adjusted versus observed BWS iNMR for the selected 26 NEST360 partnering hospitals (the blue lines show adjusted estimates and black dots show observed data)

Based on the conservative cut-off of iNMR ≥ 700/1000, 26 hospitals were identified across the four countries as having more plausible birthweight-specific in-patient mortality rates for neonates weighing < 1000 g. Figure 3c and d show the unadjusted admissions and birthweight-specific mortality rates for these 26 hospitals. Posterior distributions for the admissions and iNMR Bayesian models (fitted on data for these selected hospitals) were adequately explored, as the trace plots showed adequate mixing. The models also showed satisfactory convergence, as all the estimated PSFRs were approximately 1. See trace plots and PSFR estimates provided in the supplementary material, eFigs. 3 and 4, and eTables 4 and 5, respectively. Further, the models showed good performance, with posterior predictive mean curves closely approximating the observed patterns across birthweight categories for both admissions and iNMRs for the 26 hospitals (Figs. 4 and 5).

#### Application of the estimated adjustment curves to all NEST360 partnering hospitals to estimate baseline iNMRs

The resulting birthweight-specific admission and iNMR curves after applying the fitted models to adjust for underreporting in all 65 NEST360 phase 1 hospitals are presented in Figs. 3e and f. The adjusted curves showed plausible patterns for all hospitals with babies weighing 2500–3500 g forming the majority of admissions, with iNMR being highest in the smallest babies < 1000 g. Using data for one hospital, we provide in the supplementary material a demonstration of how these fitted models were applied to adjust for underreporting of birthweight-specific admissions and iNMRs (see eTables 6 and 7, and 8). For the four countries, the average adjusted iNMRs were plausibly higher than unadjusted estimates: 162 deaths per 1000 admissions in Nigeria 183 per 1000 in Tanzania 174 per 1000 in Malawi and 172 per 1000 in Kenya. In addition, heterogeneity between hospitals was reduced with adjusted iNMRs within countries ranging from 120 to 333 per 1000 (Table 3).

#### Neonatal Mortality Estimates based on a threshold of iNMR ≥ 750/1000 for babies < 1000 g

Twenty-two hospitals had an iNMR ≥ 750 per 1000 among babies weighing < 1000 g (Supplementary eFigure 5). Data from these hospitals were used to fit the adjustment functions, with goodness of fit (based on posterior predictive estimates) presented in Supplementary eFigures 6 and 7. Hospital-level curves derived from these adjustment functions are shown in Supplementary eFigure 8. These curves display patterns similar to those obtained using a threshold of iNMR ≥ 700 per 1000. However, the adjusted hospital-level iNMR spans a slightly wider range, from 120 to 374 per 1000, compared with 120 to 333 per 1000 when using the iNMR ≥ 700 per 1000 cut-off. Across the four countries, the adjusted iNMR estimates were slightly higher than those reported in Table 3. Using the higher threshold of iNMR ≥ 750 per 1000, estimates ranged from 165 per 1000 in Nigeria to 186 per 1000 in Tanzania (Supplementary eTable 9).

## Discussion

Our study addresses the critical issue of underreporting in-patient neonatal mortality, especially among the underweight neonates, through the development and application of birthweight-specific adjustment curves. Using simulations as a proof of concept, we demonstrated that birthweight adjustment functions robustly adjust for underreporting and enhance the plausibility of iNMR estimates. Simulated data showed good alignment between adjusted and original simulated values across all birthweight categories. For example, in the < 1000 g category, the adjusted iNMR (825 per 1000 admissions was nearly identical to the true simulated value (827 per 1000 admissions), while adjusting for the underreported figure (573 per 1000 admissions). These findings validate the strategy of the adjustment approach and establish a solid foundation for its application to real-world datasets. By anchoring estimates to biologically plausible mortality patterns, this approach offers a practical and internally consistent strategy for recalibrating in-patient neonatal mortality rates.

Within the large, multi-country NEST360 dataset, we found substantial underreporting in routine hospital-level data. Unadjusted country level iNMRs ranged from 121/1000 in Nigeria to 135/1000 in Kenya, which increased to more plausible figures – ranging from 162/1000 in Nigeria to 183/1000 in Tanzania – after adjustment. This process not only addressed underreporting but also reduced variability among hospitals, particularly for lower birthweight categories (< 2500 g), where mortality burden is highest. For example, one hospital reported iNMR of 0/1000 for neonates < 1000 g, which was adjusted to a level consistent with known survival probabilities in similar resource-limited settings. Notably, the NIH Global Network reported iNMRs exceeding 800 per 1000 for neonates < 1000 g without neonatal intensive care [38]. Similar estimates from Ghana, where preterm survival with CPAP yielded iNMRs of 720 per 1000, further substantiate the plausibility of our adjustments [39].

Our methodology offers distinct advantages over prediction-based approaches. While some studies use regression models incorporating socioeconomic or health system factors – or apply generic adjustment factors derived from expert consensus – these methods often lack contextual specificity and rely on external assumptions. In addition, widely used data sources such as DHS or vital registration systems may be affected by systemic biases, including recall error, misclassification of birthweight, and incomplete capture of early neonatal deaths, particularly in low-resource settings. In contrast, our use of birthweight-specific curves and actual hospital-level data grounds the adjustments in local realities, reducing reliance on external assumptions and mitigating biases inherent in population-based or administrative datasets. This is particularly important when mortality data are used for programme evaluation, benchmarking progress, or identifying outlier hospitals for targeted support [40].

Importantly, the application of this approach provides insight not only into mortality burdens but also into the distribution and plausibility of neonatal admissions by birthweight. Hospitals with missing or implausible data – such as very low iNMRs in high-risk birthweight categories – can be flagged, and their data adjusted using adjustment functions. For example, 21 of the 65 hospitals initially reported iNMRs below 100 per 1000 admissions, which is incongruent with current inpatient neonatal survival trends in sub-Saharan Africa [41]. After adjustment, these figures aligned better with regional inpatient epidemiology, and the revised estimates highlighted the real burden faced by neonatal units.

These findings have important policy implications. Reliable neonatal mortality data are essential for evaluating care quality, guiding resource allocation, and advocating for better investments in neonatal services. However, achieving data completeness and reliability remains a major challenge in many health systems. Policymakers should prioritise investment in data quality initiatives within systems such as DHIS2 and Electronic Medical Record Systems. This includes health worker training, routine audits, and the implementation of standardised reporting protocols. Beyond technical solutions, promoting a culture of accountability around data collection and use is crucial. Incentivising high-quality data reporting and fostering data-driven decision-making can significantly enhance both care delivery and health outcomes [42–44].

Some limitations warrant consideration. Our adjustment approach assumes that the subset of “certainty” hospitals – those with iNMR ≥ 700 per 1000 for neonates < 1000 g – reflect more complete reporting. While this threshold is supported by prior evidence, it remains somewhat arbitrary and could be refined as more granular data emerge. Additionally, while the methodology performs well for lower birthweight categories, further refinement may be needed to improve plausibility checks for higher birthweight groups, where baseline mortality is lower and underreporting may be subtler.

In conclusion, this study demonstrates the efficacy of birthweight-specific adjustment curves in addressing neonatal mortality underreporting, validated through both simulation and real-world application. By enhancing the plausibility and comparability of iNMR estimates, this approach offers a scalable and adaptable framework for improving the quality and plausibility of routine neonatal data reported in low-resource settings. However, adjustment methods should be regarded as an interim solution rather than a substitute for high-quality routine data. The overriding priority remains to strengthen health information systems to ensure that every newborn is identified, recorded and counted, including the smallest and sickest newborns who are at greatest risk of death. Improving the completeness and quality of routine data will enable more accurate measurement of neonatal outcomes, better inform quality improvement efforts, and support accountability for reducing preventable newborn deaths. Ultimately, achieving the goal to "count every newborn" is fundamental to ensuring that every newborn receives the care they need and that no newborn is left invisible in routine health information systems [45].

## Supplementary Information

Supplementary Material 1

## Data Availability

Data sharing and transfer agreements were jointly developed and signed by all collaborating partners. The data may be available upon request and approval by the NEST360 leadership.
