# Supplementary material for: Neonatal admissions and mortality: a statistical adjustment approach using birthweight-specific curves to address bias due to underreporting in 65 hospitals in four African countries
Source: Popul Health Metr. 2026 Jul 28;24:62. doi: 10.1186/s12963-026-00496-z (PMC13693040; doi:10.1186/s12963-026-00496-z)
Supplement: Supplementary file 1 [file 12963_2026_496_MOESM1_ESM.docx]

**A statistical approach to minimise bias due to undercounting of inpatient neonatal mortality in four African countries: Adjustment using birthweight-specific curves**

A) **Summary of mortality data by birthweight**

There is limited published research on birthweight-specific inpatient mortality, particularly in LMICs. The earliest review on this topic was conducted by Blencowe et al. in 2013. Since then, individual studies have examined birthweight-specific inpatient neonatal mortality rates (NMRs). The most recent systematic review was published in 2020. In this study, we synthesise and summarise the median NMRs reported across these publications. Detailed information on the studies included in this synthesis is provided in **eTable 1**.

**eTable 1**: Descriptive studies on inpatient neonatal mortality with similar contexts/care to NEST360 – reporting birthweight categories

| **Birthweight** | **Country** | **Context/Intervention** | **Baseline NMR** | **Ref, Year** |
| --- | --- | --- | --- | --- |
| **<1000gm**  **<28 weeks:** | Global review for WHO estimates all LMIC regions | Neonatal v limited care  Neonatal special care | 990/1000  864/1000 | Blencowe H, Lawn  et al. 2013 |
| **1000-1499g**  **28 – 31 weeks:** |  | Neonates with limited care  Neonatal special care | 610/1000  413/1000 |  |
| **Lancet Small Vulnerable Newborns datasets**  Subnational, population-based birth cohorts for 541 285 live births in 23 countries, 2000–2021  Neonatal mortality risk for vulnerable newborn types in 15 countries using 125.5 million nationwide birth outcome records, 2000–2020 | LMIC regions | Neonatal special care | details vary by region and BW – in process of adding | Hazeal, Black et al with Ohuma and Lawn |
|  | High and middle income | NICU | To use as lowest plausible limit for BSWM | Suarez e al with Homa and Lawn |
| **<1000g**  **1000-1499g**  **1500 - 1999g**  **2000-2499g:**  **2500-3999g** | Kenya, 16 units with CIN | Neonatal special care plus CPAP | 790/1000  430/1000  140/1000  90/1000  60/1000 | Irimu et. al, 2021 |

We summarised median birthweight specific NMRs and presented these in **eTable 2**. The summarised data indicate a strong inverse relationship between birthweight and mortality, with the highest risks observed among the smallest neonates and a gradual decline as birthweight increases, although neonates with excessive birthweight also face heightened risks due to perinatal complications.

Neonates born with a birthweight below 1000 grams, typically corresponding to a gestational age of less than 28 weeks, face exceptionally high mortality risks. The median NMR in this category is 827 per 1000 live births, with a range between 500 and 990. These neonates, classified as extremely preterm, encounter significant challenges, including respiratory distress syndrome, sepsis, and intraventricular haemorrhage. Despite advances in neonatal care, survival remains limited, particularly in resource-constrained settings where access to intensive support is restricted.

As birthweight increases to the 1000–1499 gram range, the risk of neonatal mortality decreases but remains substantial. The median NMR is 422 per 1000 live births, ranging from 250 to 610. This group consists mostly of very preterm neonates who often require prolonged respiratory support, intravenous nutrition, and careful thermoregulation. While survival rates improve with high-quality neonatal intensive care, these neonates remain highly vulnerable in the absence of specialised interventions.

Further reductions in neonatal mortality are observed among infants weighing 1500 to 1999 grams, where the median NMR is 180 per 1000 live births, with a range of 140 to 230. This category largely includes late preterm neonates, who may still face challenges such as feeding difficulties, hypothermia, and an increased risk of infection. However, their survival rates are markedly better than those of lower birthweight groups, particularly in well-equipped neonatal units.

Among neonates weighing 2000 to 2499 grams, the mortality risk declines further, with a median NMR of 140 per 1000 live births and a lower bound of 75 per 1000. Many of these neonates are either late preterm or small for gestational age. Although their overall prognosis is better, they remain at risk for complications such as hypoglycaemia and neonatal infections, particularly in settings where postnatal monitoring is inadequate.

The lowest neonatal mortality rates are observed in neonates with birthweights between 2500 and 3999 grams, the range generally associated with term births. The median NMR in this category is 90 per 1,000 live births, with a lower limit of 26 per 1,000. This birthweight range represents optimal fetal growth and maturity, resulting in the highest survival rates and the lowest risk of severe neonatal complications.

However, among neonates with birthweights exceeding 4,000 grams, mortality risks begin to rise again. While their overall NMR remains low, they face a 1.5 times higher relative risk of mortality compared to infants in the 2,500–3,999 gram range. This increase in risk is often associated with complications such as birth asphyxia, delivery trauma due to macrosomia, and metabolic disorders like neonatal hypoglycaemia.

**eTable 2:** Median NMR per birthweight category summarised from published literature

| **Birthweight**  **[approx. GA without SGA]** | **NMR per 1000 median**  **[range]** |
| --- | --- |
| <1000gm  [~<28 weeks] | 827  [500-990] |
| 1000-1499g | 422  [250-610] |
| 1500 - 1999g | 180  [140-230] |
| 2000 – 2499g | 140  [75 lower limit] |
| 2500-3999g | 90  [26 lower limit] |
| **>4000g** | **>4500 1.5 x RR compared to normal BWT** |

B) **Summaries from simulations**

**eTable 3: Summary of simulated admissions and mortality across 1000 facilities**

| **Birthweight** | **Admissions** | | | **Mortality per 1000** | | |
| --- | --- | --- | --- | --- | --- | --- |
|  | Median | Min | Max | Median | Min | Max |
| 0–1000g | 57 | 28 | 84 | 827 | 671 | 911 |
| 1001–1500g | 78 | 43 | 117 | 420 | 329 | 520 |
| 1501–2000g | 136 | 96 | 190 | 178 | 91 | 286 |
| 2001–2500g | 220 | 176 | 273 | 137 | 63 | 241 |
| 2501–3000g | 263 | 214 | 323 | 85 | 20 | 219 |
| 3001–3500g | 221 | 170 | 270 | 83 | 14 | 230 |
| 3501–4000g | 137 | 101 | 185 | 78 | 16 | 209 |
| 4001–4500g | 78 | 45 | 115 | 116 | 43 | 232 |
| 4501–5000g | 55 | 29 | 92 | 126 | 55 | 253 |

**eFigure 1:** Distribution of adjusted versus observed BWS admission numbers for selected 30 facilities (the blue lines show fitted estimates and black dots show simulated data).

**eFigure 2:** Distribution of adjusted versus observed BWS admission numbers for selected 30 facilities (the blue lines show fitted estimates and black dots show simulated data).

B) **Diagnostics and model outputs considering threshold of NMR>=700/1000 for babies < 1000g**

**History plots of sampled estimates (examining how posterior distributions were explored)**

**eFigure 3: Admission Models**

3.1. Birthweight < 1000g

3.2. Birthweight 1001 – 1500g

3.3. Birthweight 1501 – 2000g

3.4. Birthweight 2001 – 2500g

3.5. Birthweight 2501 – 3000g

3.6. Birthweight 3001 – 3500g

3.7. Birthweight 3501 – 4000g

3.8. Birthweight 4001 – 4500g

3.9. Birthweight 4501+ g

**eFigure 4: Admission Models**

4.1. Birthweight < 1000g

4.2. Birthweight 1001 – 1500g

4.3. Birthweight 1501 – 2000g

4.4. Birthweight 2001 – 2500g

4.5. Birthweight 2501 – 3000g

4.6. Birthweight 3001 – 3500g

4.7. Birthweight 3501 – 4000g

4.8. Birthweight 4001 – 4500g

4.9. Birthweight 4501+ g

5. **Fitted model estimates (95% Credible Intervals, effective sample and PFSR)**

**eTable 4**: Admission models

|  | **mean estimate** | **2.50%** | **97.50%** | **Effective sample size** | **PSFR** |
| --- | --- | --- | --- | --- | --- |
| Model for `<1000` | | | | | |
| (Intercept) | 3.329 | 2.915 | 3.786 | 36689.083 | 1.000 |
| `1001-1500` | 0.003 | -0.002 | 0.007 | 18831.651 | 1.000 |
| `1501-2000` | 0.001 | -0.003 | 0.005 | 24107.675 | 1.000 |
| `2001-2500` | 0.000 | -0.001 | 0.001 | 28034.855 | 1.000 |
| `2501-3000` | 0.000 | -0.002 | 0.002 | 23026.695 | 1.000 |
| `3001-3500` | -0.001 | -0.003 | 0.001 | 17857.070 | 1.000 |
| `3501-4000` | 0.002 | -0.002 | 0.005 | 20308.293 | 1.000 |
| `4001-4500` | 0.001 | -0.003 | 0.005 | 22800.231 | 1.000 |
| `4501+` | -0.004 | -0.011 | 0.004 | 26376.013 | 1.000 |
| Model for `1001-1500` | | | | | |
| (Intercept) | 4.609 | 4.200 | 5.032 | 36193.451 | 1.000 |
| `<1000` | 0.000 | -0.004 | 0.004 | 25559.552 | 1.000 |
| `1501-2000` | 0.003 | 0.000 | 0.005 | 25743.032 | 1.000 |
| `2001-2500` | 0.000 | -0.001 | 0.001 | 29005.786 | 1.000 |
| `2501-3000` | 0.000 | -0.002 | 0.002 | 23968.763 | 1.000 |
| `3001-3500` | -0.001 | -0.003 | 0.001 | 19040.697 | 1.000 |
| `3501-4000` | 0.000 | -0.003 | 0.003 | 20340.034 | 1.000 |
| `4001-4500` | 0.001 | -0.002 | 0.004 | 27287.243 | 1.000 |
| `4501+` | 0.000 | -0.007 | 0.008 | 26313.544 | 1.000 |
| Model for `1501-2000` | | | | | |
| (Intercept) | 5.288 | 4.904 | 5.657 | 32789.563 | 1.000 |
| `<1000` | 0.000 | -0.004 | 0.004 | 27763.892 | 1.000 |
| `1001-1500` | 0.002 | -0.001 | 0.005 | 25054.916 | 1.000 |
| `2001-2500` | 0.000 | 0.000 | 0.001 | 29366.979 | 1.000 |
| `2501-3000` | 0.000 | -0.002 | 0.002 | 20253.473 | 1.000 |
| `3001-3500` | 0.000 | -0.002 | 0.002 | 17465.429 | 1.000 |
| `3501-4000` | 0.000 | -0.004 | 0.003 | 20107.032 | 1.000 |
| `4001-4500` | -0.001 | -0.004 | 0.002 | 22188.833 | 1.000 |
| `4501+` | 0.002 | -0.006 | 0.010 | 24237.389 | 1.000 |
| Model for `2001-2500` | | | | | |
| (Intercept) | 5.135 | 4.652 | 5.627 | 40348.683 | 1.000 |
| `<1000` | -0.001 | -0.006 | 0.004 | 30171.456 | 1.000 |
| `1001-1500` | -0.002 | -0.007 | 0.003 | 25509.129 | 1.000 |
| `1501-2000` | 0.003 | 0.000 | 0.006 | 26555.790 | 1.000 |
| `2501-3000` | 0.002 | 0.000 | 0.004 | 29058.007 | 1.000 |
| `3001-3500` | -0.001 | -0.004 | 0.001 | 20944.541 | 1.000 |
| `3501-4000` | 0.000 | -0.004 | 0.004 | 21640.698 | 1.000 |
| `4001-4500` | 0.001 | -0.003 | 0.004 | 23722.009 | 1.000 |
| `4501+` | 0.001 | -0.008 | 0.010 | 28114.326 | 1.000 |
| Model for `2501-3000` | | | | | |
| (Intercept) | 5.677 | 5.205 | 6.138 | 42448.220 | 1.000 |
| `<1000` | 0.000 | -0.005 | 0.005 | 27890.515 | 1.000 |
| `1001-1500` | 0.001 | -0.004 | 0.005 | 22588.570 | 1.000 |
| `1501-2000` | 0.000 | -0.004 | 0.003 | 20224.754 | 1.000 |
| `2001-2500` | 0.001 | 0.000 | 0.002 | 33035.246 | 1.000 |
| `3001-3500` | 0.001 | -0.001 | 0.002 | 17204.991 | 1.000 |
| `3501-4000` | -0.001 | -0.004 | 0.002 | 17516.991 | 1.000 |
| `4001-4500` | 0.000 | -0.003 | 0.003 | 22057.570 | 1.000 |
| `4501+` | 0.001 | -0.006 | 0.010 | 26389.666 | 1.000 |
| Model for `3001-3500` | | | | | |
| (Intercept) | 5.953 | 5.493 | 6.401 | 41043.973 | 1.000 |
| `<1000` | 0.000 | -0.004 | 0.005 | 36734.465 | 1.000 |
| `1001-1500` | 0.000 | -0.004 | 0.004 | 28543.739 | 1.000 |
| `1501-2000` | 0.000 | -0.004 | 0.003 | 29427.752 | 1.000 |
| `2001-2500` | 0.000 | -0.001 | 0.001 | 34769.460 | 1.000 |
| `2501-3000` | 0.001 | -0.001 | 0.002 | 23391.211 | 1.000 |
| `3501-4000` | 0.001 | -0.001 | 0.002 | 24476.616 | 1.000 |
| `4001-4500` | -0.001 | -0.004 | 0.003 | 29380.338 | 1.000 |
| `4501+` | 0.003 | -0.005 | 0.011 | 27149.507 | 1.000 |
| Model for `3501-4000` | | | | | |
| (Intercept) | 5.073 | 4.602 | 5.578 | 40783.009 | 1.000 |
| `<1000` | 0.001 | -0.003 | 0.005 | 40124.501 | 1.000 |
| `1001-1500` | 0.001 | -0.004 | 0.006 | 27392.934 | 1.000 |
| `1501-2000` | -0.001 | -0.005 | 0.002 | 33141.657 | 1.000 |
| `2001-2500` | 0.000 | -0.001 | 0.002 | 34441.663 | 1.000 |
| `2501-3000` | -0.001 | -0.003 | 0.001 | 25500.648 | 1.000 |
| `3001-3500` | 0.001 | 0.000 | 0.002 | 26225.356 | 1.000 |
| `4001-4500` | 0.001 | -0.003 | 0.004 | 28013.202 | 1.000 |
| `4501+` | 0.004 | -0.005 | 0.012 | 23402.333 | 1.000 |
| Model for `4001-4500` | | | | | |
| (Intercept) | 4.039 | 3.397 | 4.695 | 44687.717 | 1.000 |
| `<1000` | 0.002 | -0.005 | 0.009 | 30189.435 | 1.000 |
| `1001-1500` | 0.001 | -0.005 | 0.008 | 27391.006 | 1.000 |
| `1501-2000` | -0.002 | -0.007 | 0.002 | 23746.488 | 1.000 |
| `2001-2500` | 0.001 | -0.001 | 0.003 | 25636.523 | 1.000 |
| `2501-3000` | 0.001 | -0.002 | 0.004 | 24273.965 | 1.000 |
| `3001-3500` | -0.001 | -0.004 | 0.002 | 18880.859 | 1.000 |
| `3501-4000` | 0.003 | -0.002 | 0.008 | 19692.397 | 1.000 |
| `4501+` | 0.007 | -0.004 | 0.018 | 27269.187 | 1.000 |
| Model for `4501+` | | | | | |
| (Intercept) | 1.914 | 1.197 | 2.605 | 43459.131 | 1.000 |
| `<1000` | -0.001 | -0.007 | 0.005 | 27095.381 | 1.000 |
| `1001-1500` | 0.004 | -0.003 | 0.011 | 21638.602 | 1.000 |
| `1501-2000` | 0.000 | -0.006 | 0.005 | 21668.699 | 1.000 |
| `2001-2500` | 0.000 | -0.002 | 0.002 | 29117.162 | 1.000 |
| `2501-3000` | -0.002 | -0.005 | 0.001 | 24805.014 | 1.000 |
| `3001-3500` | -0.001 | -0.004 | 0.003 | 15471.162 | 1.000 |
| `3501-4000` | 0.004 | -0.001 | 0.009 | 14830.909 | 1.000 |
| `4001-4500` | 0.006 | 0.001 | 0.010 | 21699.651 | 1.000 |

**eTable 5**: NMR models

|  | **log OR** | **2.50%** | **97.50%** | **Effective Sample Size** | **PSFR** |
| --- | --- | --- | --- | --- | --- |
| Model for `<1000` | | | | | |
| (Intercept) | 0.539 | -0.267 | 1.336 | 55852.007 | 1.000 |
| `1001-1500` | 2.173 | -0.859 | 5.293 | 30091.585 | 1.000 |
| `1501-2000` | -0.216 | -9.099 | 8.699 | 27026.194 | 1.000 |
| `2001-2500` | -0.830 | -7.569 | 6.134 | 31610.120 | 1.000 |
| Model for `1001-1500` | | | | | |
| (Intercept) | -3.187 | -5.845 | -0.540 | 38868.849 | 1.000 |
| `<1000` | 2.426 | -1.121 | 5.970 | 35796.721 | 1.000 |
| `1501-2000` | 5.873 | -0.827 | 12.628 | 26950.140 | 1.000 |
| `2001-2500` | -0.117 | -6.408 | 5.898 | 28875.402 | 1.000 |
| Model for `1501-2000` | | | | | |
| (Intercept) | -2.378 | -5.514 | 0.940 | 34358.836 | 1.000 |
| `<1000` | -0.304 | -5.011 | 4.179 | 29772.326 | 1.000 |
| `1001-1500` | 1.531 | -1.423 | 4.400 | 26422.621 | 1.000 |
| `2001-2500` | 2.932 | -3.035 | 8.653 | 33403.105 | 1.000 |
| Model for `2001-2500` | | | | | |
| (Intercept) | -2.378 | -5.932 | 1.349 | 32837.056 | 1.000 |
| `<1000` | -0.642 | -6.033 | 4.533 | 30177.268 | 1.000 |
| `1001-1500` | 0.061 | -4.045 | 3.967 | 25763.334 | 1.000 |
| `1501-2000` | 6.228 | -2.688 | 14.560 | 27133.187 | 1.000 |
| Model for `2501-3000` | | | | | |
| (Intercept) | -2.797 | -3.414 | -2.183 | 39514.447 | 1.000 |
| `3001-3500` | 10.216 | -2.861 | 23.594 | 23264.266 | 1.000 |
| `3501-4000` | -0.827 | -13.634 | 11.610 | 24089.591 | 1.000 |
| `4001-4500` | -0.707 | -6.597 | 4.907 | 38384.081 | 1.000 |
| `4501+` | -1.172 | -6.637 | 3.835 | 36474.620 | 1.000 |
| Model for `3001-3500` | | | | | |
| (Intercept) | -2.967 | -3.618 | -2.313 | 46585.027 | 1.000 |
| `2501-3000` | 5.398 | -3.169 | 13.915 | 30530.525 | 1.000 |
| `3501-4000` | 2.772 | -7.766 | 12.880 | 28352.504 | 1.000 |
| `4001-4500` | 0.813 | -4.927 | 6.353 | 40077.589 | 1.000 |
| `4501+` | -0.248 | -6.193 | 5.015 | 33575.098 | 1.000 |
| Model for `3501-4000` | | | | | |
| (Intercept) | -3.020 | -3.702 | -2.332 | 36838.935 | 1.000 |
| `2501-3000` | 1.349 | -12.186 | 14.584 | 20272.982 | 1.000 |
| `3001-3500` | 7.257 | -9.352 | 24.072 | 18828.813 | 1.000 |
| `4001-4500` | -0.222 | -6.841 | 6.131 | 28899.246 | 1.000 |
| `4501+` | 0.490 | -5.460 | 5.766 | 27388.179 | 1.000 |
| Model for `4001-4500` | | | | | |
| (Intercept) | -3.166 | -4.086 | -2.248 | 33999.427 | 1.000 |
| `2501-3000` | -1.135 | -18.167 | 15.337 | 24474.753 | 1.000 |
| `3001-3500` | 20.139 | -5.119 | 45.140 | 22503.102 | 1.000 |
| `3501-4000` | -16.517 | -33.690 | 0.183 | 27117.782 | 1.000 |
| `4501+` | 5.083 | -1.967 | 11.687 | 29406.237 | 1.000 |
| Model for `4501+` | | | | | |
| (Intercept) | -3.522 | -4.696 | -2.356 | 33660.946 | 1.000 |
| `2501-3000` | 1.654 | -17.634 | 20.714 | 25702.247 | 1.000 |
| `3001-3500` | -1.252 | -31.269 | 28.678 | 22601.107 | 1.000 |
| `3501-4000` | 3.824 | -12.484 | 20.051 | 30222.874 | 1.000 |
| `4001-4500` | 2.119 | -5.434 | 9.393 | 33124.438 | 1.000 |

6. **Illustrating how the models are used for adjustments**

We illustrate how we used the estimated models to adjust for underreporting; using data for one facility which reported the following distributions by birthweight category.

**eTable 6**: Birthweights specific NMR and admissions for selected facility example

| Birthweight category (grams) | Unadjusted NMR (proportion) | Unadjusted Admissions |
| --- | --- | --- |
| <1000 | 0.000 | 1 |
| 1001 - 1500 | 0.200 | 25 |
| 1501 - 2000 | 0.030 | 33 |
| 2001 - 2500 | 0.020 | 49 |
| 2501 - 3000 | 0.022 | 92 |
| 3001 - 3500 | 0.035 | 57 |
| 3501 - 4000 | 0.000 | 17 |
| 4000 - 4500 | 0.000 | 5 |
| 4501+ | 0.000 | 0 |

We only show admission and NMR adjustment for babies weighing less than 1000g. **eTable 6** presents the predicted admission numbers and percentages for each birthweight category across the 26 facilities selected for curve fitting.

**eTable 7**: Distribution of predicted admission numbers from the 26 facilities selected for curve fitting

| **Model** | **Predicted admission numbers** | **Proportion** |
| --- | --- | --- |
| <1000 | 2393 | 0.02 |
| 1001 - 1500 | 7419 | 0.07 |
| 1501 - 2000 | 12390 | 0.12 |
| 2001 - 2500 | 14358 | 0.14 |
| 2501 - 3000 | 21405 | 0.21 |
| 3001 - 3500 | 25342 | 0.25 |
| 3501 - 4000 | 12561 | 0.12 |
| 4000 - 4500 | 4070 | 0.04 |
| 4501+ | 1670 | 0.02 |
| **Total** | 101608 | **1.00** |

The facility example has lower admission proportions in the following birthweight categories: <1000g, 3001–3500g, 3501–4000g, 4000–4500g, and 4501+g (see **eTable 8**). Admission data for these six birthweight categories should be adjusted. However, for demonstration purposes, we illustrate the adjustment for birthweight <1000g.

**eTable 8**: Comparing distribution of admissions between the fitted data from 26 facilities versus the distribution in facility example

| **Model** | **Expected admission distribution based on 26 facilities used for curve fitting** | **Distribution for example facility above** |
| --- | --- | --- |
| <1000 | 0.02 | 0.00 |
| 1001 - 1500 | 0.07 | 0.09 |
| 1501 - 2000 | 0.12 | 0.12 |
| 2001 - 2500 | 0.14 | 0.18 |
| 2501 - 3000 | 0.21 | 0.33 |
| 3001 - 3500 | 0.25 | 0.20 |
| 3501 - 4000 | 0.12 | 0.06 |
| 4000 - 4500 | 0.04 | 0.02 |
| 4501+ | 0.02 | 0.00 |

- For babies <1000g, the adjusted admissions would be computed as follows:

0.02 (anticipated proportion for <1000g) x 279 (Total admissions for facility example)

= 0.02 x 279 = 5.58

- From eTable 5, the mortality model for babies < 1000g becomes:

$logit({NMR}_{BWT=<1000g})=$0.539 + ${(BWT}_{`1001-1500 NMR`}\times2.173)$+ (${BWT}_{`1501-2000 NMR`}\times-0.216)+ {(BWT}_{`2001-2500 NMR`}\times-0.830)$

Applying observed NMR data for this facility to the formula, we have:

$logit\left( {NMR}_{BWT=<1000g} \right)=$0.539 + $\left( 0.200\times2.173 \right)$+ ($0.030\times-0.216)+(0.020\times-0.830)=0.95052$

The inverse logit of 0.95052 is 0.7212. This implies that the adjusted mortality in this facility is approximately 721/1000.

C) **Model outputs considering threshold of NMR>=750/1000 for babies < 1000g**

**eFigure 5**: Distribution of admissions and BWSM rate for NEST360 facilities with plausible BWSM rate of >=750/1000 in neonates with birthweight <1000g across the four countries (N=22 neonatal units)

**eFigure 6**: Distribution of adjusted versus observed BWS admission numbers for the selected 22 NEST360 neonatal units (the blue lines show adjusted estimates and black dots show observed data).

**eFigure 7**: Distribution of adjusted versus observed BWS NMR for the selected 22 NEST360 neonatal units (the blue lines show adjusted estimates and black dots show observed data).

**eFigure 8**: Distribution of adjusted admission and BWSM rates (NMR ≥ 750/1000 threshold) for all NEST360 facilities (N=65 facilities)

**eTable 9**: Adjusted baseline NMR estimates for 65 NEST360 neonatal units by country

(Based on NMR ≥ 750/1000 threshold)

| **Country** | **Adjusted NMR/1000 admissions**  **(facility range)** | **Number of facilities with NMR (per 1000)** | | | |
| --- | --- | --- | --- | --- | --- |
|  |  | **> 200** | **>150 – 200** | **>100 – 150** | **<=100** |
| Kenya | 174 [124 – 264] | 4 | 5 | 4 | 0 |
| Malawi | 177 [120 – 278] | 9 | 18 | 9 | 0 |
| Nigeria | 165 [124 – 374] | 1 | 5 | 3 | 0 |
| Tanzania | 186 [146 – 235] | 2 | 2 | 3 | 0 |
| **Total** | **177 [120 – 374]** | **16** | **30** | **19** | **0** |
